# Electronic Health Record Stress and Burnout Among Clinicians in Hospital Settings: A Systematic Review

**DOI:** 10.1177/20552076231220241

**Published:** 2023-12-19

**Authors:** Fatimah Alobayli, Siobhan O’Connor, Aisha Holloway, Kathrin Cresswell

**Affiliations:** 1 Nursing Studies, School of Health in Social Science, 215746 The University of Edinburgh, Edinburgh, UK; 2 Division of Nursing, Midwifery and Social Work, School of Health Sciences, 5292 The University of Manchester, Manchester, UK; 3 College of Medicine and Veterinary Medicine, Usher Institute, 572451 The University of Edinburgh, Edinburgh, UK

**Keywords:** Systematic review, electronic health records, burnout, stress, clinicians, hospital

## Abstract

**Background:**

There is growing evidence to suggest that EHRs may be associated with clinician stress and burnout, which could hamper their effective use and introduce risks to patient safety.

**Objective:**

This systematic review aimed to examine the association between EHR use and clinicians’ stress and burnout in hospital settings, and to identify the contributing factors influencing this relationship.

**Methods:**

The search included peer-reviewed published studies between 2000 and 2023 in English in CINAHL, Ovid Medline, Embase, and PsychINFO. Studies that provided specific data regarding clinicians’ stress and/or burnout related to EHRs in hospitals were included. A quality assessment of included studies was conducted.

**Results:**

Twenty-nine studies were included (25 cross-sectional surveys, one qualitative study, and three mixed methods), which focused on physicians (n = 18), nurses (n = 10) and mixed professions (n = 3). Usability issues and the amount of time spent on the EHR were the most significant predictors, but intensity of the working environment influenced high EHR-related workload and thereby also contributed to stress and burnout. The differences in clinicians’ specialties influenced the levels of stress and burnout related to EHRs.

**Conclusions:**

This systematic review showed that EHR use was a perceived contributor to clinicians’ stress and burnout in hospitals, primarily driven by poor usability and excessive time spent on EHRs. Addressing these issues requires tailored EHR systems, rigorous usability testing, support for the needs of different specialities, qualitative research on EHR stressors, and expanded research in Non-Western contexts.

## Introduction

Frontline clinicians, encompassing roles such as physicians and nurses, experience elevated levels of stress and burnout compared to the general population.^1,2^ For instance, Shanafelt et al.^1^ identified that among physicians specifically, those at the frontline of care access, reported burnout rates exceeding 50%. Meanwhile, a survey revealed high rates of burnout among nurses working in hospitals across Europe and the United States, which has a direct influence on care quality and patient safety.^3^ Burnout is defined as the final stage of chronic stress that occurs among employees and is characterised by emotional exhaustion, depersonalisation, and a lack of a sense of personal accomplishment.^4,5^ Several studies have identified associations between clinician burnout and low-quality patient care, the increased risk of medical errors, patient dissatisfaction and poor outcomes, low productivity, job dissatisfaction, sick leave, absences, turnover, or early retirement,^6–8^ all of which adversely affect healthcare costs.^7,9^ Among physicians, burnout is also linked to low resilience, substance abuse,^7,10^ poor self-care, and suicidal ideation.^7^ Many factors contribute to clinicians’ stress and burnout, including busy work environments, lack of value alignment between leaders and healthcare workers, time and productivity pressure, excessive bureaucratic tasks, and the increasing computerisation of clinical practice.^6,7^

Electronic health records (EHRs) are increasingly being implemented worldwide to improve healthcare quality, safety, and efficiency. They are widely seen as a way to tackle key issues in healthcare to achieve the ‘Triple Aim’ established by the Institute for Healthcare Improvement (IHI) in order to improve patient experience of care and population health and reduce healthcare costs.^11,12^ However, the widespread implementation of EHRs has produced unintended consequences, such as increasing the clerical burden by altering patient-provider interaction and may distract from what providers and patients perceive as meaningful aspects of healthcare practice.^13–19^ This has weakened the effective use of EHRs and introduced risks to clinicians’ wellbeing, which threatened the success of IHI goals.^12^ Recent studies reveal that the use of EHRs is one of the main factors that contribute to clinician burnout in developed Western countries, which has become a phenomenon in healthcare and has been identified as requiring further attention.^12,20,21^ The growing evidence on these topics has led leaders and policymakers to advocate for reforming the IHI model introducing a Quadruple Aim that focuses on clinicians’ wellbeing.^12^

The existing literature has provided some insights and understanding in this area. From existing reviews, the focus has been on burnout solely among clinicians, where the EHR was one of the identified contributing factors.^22,23^ The association between EHR use and clinicians’ wellbeing has also been the focus of attention recently.^24–26^ However, these reviews focused on a range of clinical settings collectively and the results were not disaggregated (i.e., hospitals from non-hospitals). Given the nature of stressful environments in hospitals, it is acknowledged that EHR-related stress and burnout can be influenced by the complexity and intensity of the working environment.^27–31^ Hence, this systematic review aimed to synthesise evidence from all types of study designs to examine the association between EHR use and clinicians’ stress and burnout in hospital settings. Additionally, it sought to identify factors contributing to clinicians’ stress and burnout when using EHR in these settings.

## Methods

### Search strategy

The search strategy was developed after consulting a specialist librarian. It was mainly tailored to four major nursing and medical databases (CINAHL, Ovid Medline, Embase, and PsychINFO). The timeframe was limited from 2000 to 2023, and only articles published in English were included. Studies conducted in hospitals prior to 2000 are unlikely to be comparable to those conducted in recent times because EHRs were developed to include more than medical records after this period, integrating clinical documentation systems for medicine, nursing, and allied healthcare.^32^

In terms of burnout, we included studies that looked at stress related to EHR use, because burnout is a result of persistent stress in the workplace.^4,5^ The search was based on key terms related to the population, intervention, and outcomes of interest i.e., burnout among clinicians using EHRs (Appendices 1 and 2). In terms of the intervention, we did not limit the search to EHRs but included technologies that supported or were integrated with EHRs. Searching for articles with specific types of study designs and settings might have limited the search. Thus, no restrictions concerning study designs and settings were applied during the database search. Medical Subject Headings (MeSH) terms, subheadings, and broad scoping searches helped extract the search terms that were used (See Appendices 1 and 2).

### Inclusion and exclusion criteria

**Figure 1.**
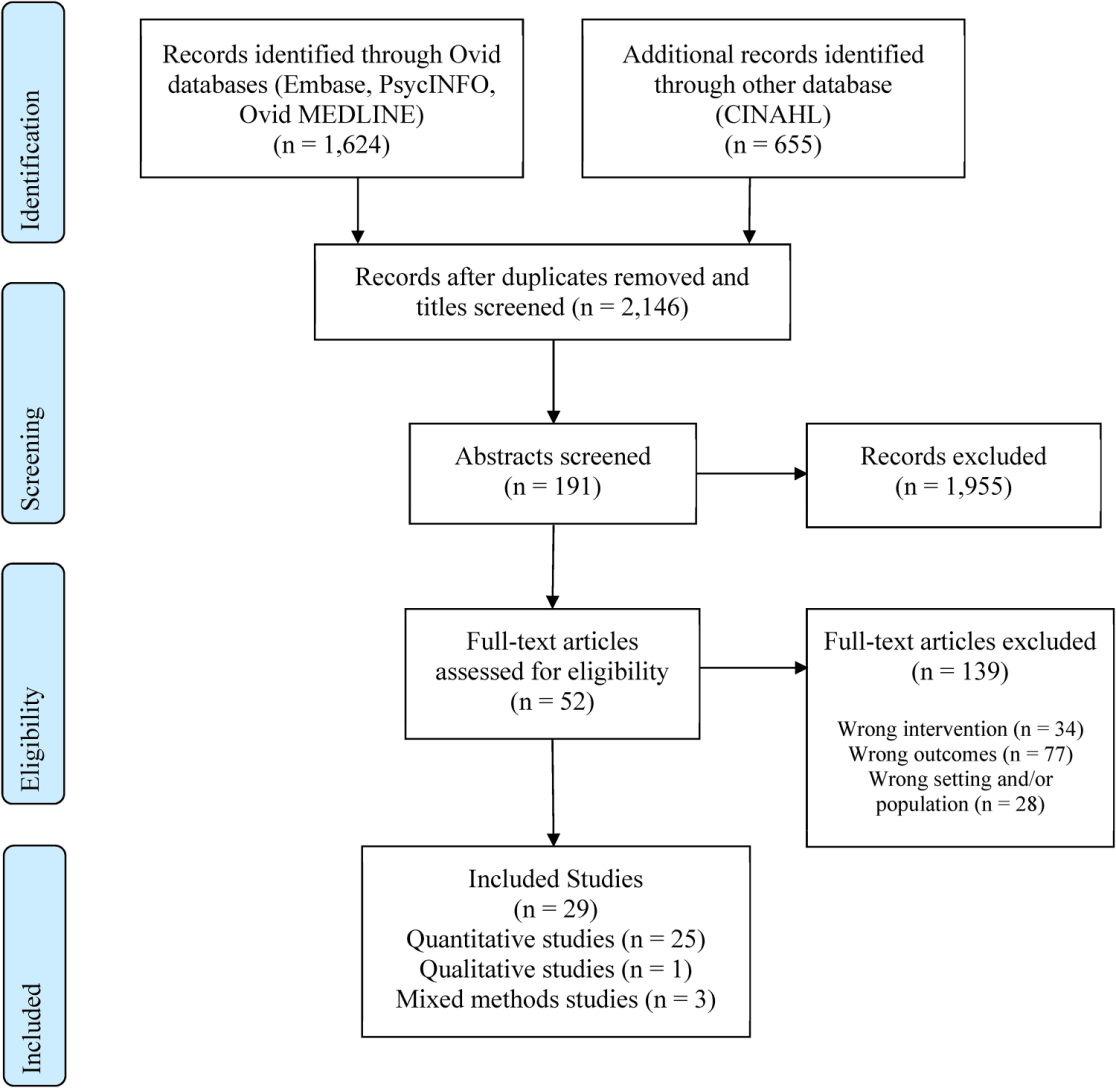
PRISMA diagram for study selection.

Articles that met the PICOSS criteria (Table 1) were included in the review. PICOSS is the modified framework for PICO, which stands for population, intervention, comparator and outcome,^33^ and includes study design and setting. PRISMA guidelines were followed to show the study selection process (Figure 1).^34^

**Table 1.** PICOSS inclusion criteria for studies.

PICOSS	Inclusion criteria	Exclusion criteria
Population	Nurses, physicians and other clinicians (such as pharmacists, physiotherapists, respiratory therapists, and midwives).	Non-clinicians, nursing or medical students, and educators.
Intervention	EHRs or technologies that include or are integrated with an EHR system.	Other types of health technology that do not support EHRs.
Comparison	None	None
Outcomes	Burnout or stress related to EHRs.	Other EHR measures that do not measure stress or burnout, such as documentation time, adoption, usability issues, barriers, dis/satisfaction, alert fatigue, burden, workload, etc.
Study design	All types of study designs involve primary data collection and analysis such as quantitative, qualitative, and mixed methods design.	Studies involving secondary data, non-empirical articles, and conference articles.
Setting	Hospital, inpatient or mixed settings.	Studies that included only outpatient, primary or community healthcare settings.

### Screening and management of studies

Endnote reference management software was used to facilitate retrieval, removal of duplicates, and manage articles. Articles identified through the search process were assessed concerning whether they met the inclusion criteria (see Table 1) and their relevance to the study topic. There were three stages to the screening process. As a first step, the researcher screened a total of 2146 article titles. In the second step, the researcher and a second reviewer independently screened abstracts for 191 articles. Finally, both the researcher and the second reviewer independently screened the full texts of the remaining studies (i.e., 52 articles). The reference lists of the remaining studies were also checked, and no additional articles were included. Information about each article's information and reviewer comments and decisions were presented in an Excel spreadsheet. A total of 29 studies were included in the review (Figure 1).

### Quality assessment of included studies

The Critical Appraisal Skills (CASP) programme checklist tools were used to assess the methodological quality of the quantitative and qualitative studies included in the review.^35^ CASP Cohort Study Checklist^36^ was used for the cross-sectional survey studies (n = 25) (Appendix 5), and CASP Qualitative Studies Checklist^37^ was used for the qualitative study (n = 1) (Appendix 4). Three mixed methods studies^14,15,38^ were assessed with the Mixed Methods Appraisal Tool (MMAT), applying the appropriate questions for the mixed methods category.^39^ Two categories of the study design in MMAT were removed from the table; randomised controlled trial and nonrandomised study design as they are not applicable to the included studies (Appendix 3). The critical appraisal was conducted by the researcher and reviewed by an expert academic researcher in nursing informatics. Quality ratings are considered high with 80%–100% of the total score per each study, moderate 50–70%, and less than 50%. Across the 29 studies, three studies were deemed to have a high risk of bias,^17,40,41^ eight had a moderate risk of bias,^14,15,38,42–46^ and 18 studies had a low risk of bias^16,47–63^ (see Appendices 3, 4 and 5). However, all the studies selected were included in the final analysis.

### Data extraction and synthesis

Data were extracted from eligible studies and summarised using Excel spreadsheets, which helped facilitate the comparison of findings, the grouping of key concepts, and the identification of the main elements to provide explanations of the findings.^64^ Data were extracted under the following headings: title of the study, name(s) of the author(s), year of publication, journal, country, aims of the study, setting, study design, participants, intervention, outcome measures, key findings, and major themes. The studies identified in this review included different study designs, different burnout measurements, and statistical heterogeneity, which made the quantitative synthesis of the empirical evidence (meta-analysis) unfeasible (Table 2). Therefore, the studies were summarised using thematic analysis to aggregate and compare findings from the included studies.^65^ The review is presented with themes that were generated from the findings of the studies using the deductive approach, drawing on the key findings that answered the review objectives (Table 2). The synthesis involved initially describing the study characteristics and the assessment methods of stress and burnout, which was then followed by a summary of the review outcomes of the EHR contribution to stress and burnout among clinicians in hospitals and the contributing factors to this issue.

**Table 2.** EHR measures, stress and/or burnout, and key themes of the reviewed studies.

	Study, year, country	Study design	Included population	Measures of general stress/burnout and EHR-specific	Key themes (EHR outcomes & influencing factors)
1	AlQahtani et al., 2020, ^40^ Saudi Arabia	Cross-sectional survey	Nurses	– EHR-related stress (10 questions)^a^	– EHR-related stress – Professional (years of experience) – Organisational (documentation) – Technological (data correction, data retrieval, training)
2	Almulhem et al., 2021,^63^ Saudi Arabia	Cross-sectional survey	Physicians, Nurses, Pharmacists, and others	– Mini-Z (single-item) – EHR measures from Mini-Z, perceptions of EHR^a^	– EHR-related burnout – EHR-related stress – Demographic (female) – Professional (speciality) – Organisational (remote access, time, tertiary level of care, COVID) – Technological (usability)
3	Anderson et al., 2022,^45^ USA	Cross-sectional survey	Gastroenterologists (physicians)	– MBI (22-item) – EHR workload and user friendliness^a^	– EHR-related burnout – Technological (usability)
4	Chen et al., 2021,^46^ China	Cross-sectional survey	Physicians	– Stress – Basic and advanced features of HIT^a^	– EHR-related stress – Technological (advanced features)
5	Elliot et al., 2022,^57^ USA	Cross-sectional survey	Physicians	– Psychological distress measures – EHR-related problems (related technical & workload issues)^a^	– EHR-related stress – Technological (usability)
6	Eschenroeder et al., 2021,^58^ USA	Cross-sectional survey	Physicians	– Mini-Z (single-item) – EHR after hour charting^a^	– EHR-related burnout – Professional (speciality) – Organisational (time after-hours) – Technological (vendor, EHR support, training, implementation)
7	Gardner et al., 2019,^47^ USA	Cross-sectional survey	Physicians	– Mini-Z (single-item) – HIT-related stress measures (frustration, time spent on EHR at home, documentation time sufficiency), perceptions about EHR^a^	– EHR-related burnout – EHR-related stress – Demographic (gender) – Organisational (time, vendors)
8	Gesner et al., 2022,^59^ USA	Cross-sectional survey	Nurses	– MBI (22-item) – EHR usability (SUS)^a^	– EHR-related burnout – Organisational (documentation) – Technological (usability)
9	Ghahramani et al., 2009,^42^ USA	Cross-sectional survey	Physicians (including fellows and residents) and Nurses	– User satisfaction, user friendliness, system familiarity, frequency of use, stress and frustration, net stress, perceptions about CPOE^a^	– EHR-related stress – Demographic (age) – Professional (speciality) – Technological (training)
10	Harris et al., 2018,^48^ USA	Cross-sectional survey	Nurses	– Mini-Z (single-item) – HIT-related stress (frustration, time spent on EHR at home, documentation time sufficiency), perceptions about EHR^a^	– EHR-related burnout – EHR-related stress – Demographic (age) – Professional (speciality) – Organisational (time)
11	Hauer et al., 2018,^41^ USA	Cross-sectional survey	Physicians	– Mini-Z (10-item), frustrations related to EHR^a^	– Burnout related to EHR – Organisational (time, work environment, workload, insurance)
12	Heponiemi et al., 2017,^49^ Finland	Cross-sectional survey	Physicians	– Stress related to HIT (2 items; constantly changing information system and IT issues)^a^	– EHR-related stress – Demographic (age) – Organisational (time pressure)
13	Jackson 2020, ^43^ USA	Cross-sectional survey	Nurses	– The Big Five Inventory (BFI) to measure technostress: techno-overload, techno-uncertainty, techno-complexity, techno-invasion, and techno-insecurity^a^	– EHR-related stress – Organisational (overload) – Technological (techno-complexity, constant change and updating)
14	Kaihlanen et al., 2021,^60^ Finland	Cross-sectional survey	Graduated (up to 2yrs) and experienced nurses (>2yrs)	– SRIS (two items), Stress, psychological distress, nursing informatics competence^a^	– EHR-related stress – Professional (years of experience) – Technological (usability)
15	Kutney-Lee et al., 2021,^50^ USA	Cross-sectional survey	Nurses	– -MBI (EE subscale) – -EHR usability^a^	– -Burnout related to EHR – -Technological (usability)
16	Marckini 2019,^17^ Canada and USA	Cross-sectional survey	Physicians	– -MBI (22-item) – -Time spent on EHR (including CPOE & patient portals), perceptions about EHR^a^	– -EHR-related burnout – -Organisational (time)
17	Melnick, Dyrbye et al., 2020,^51^ USA	Cross-sectional survey	Physicians	– MBI (EE & DP) – EHR usability (SUS)^a^	– EHR-related burnout – Demographic (gender) – Professional (speciality)
18	Melnick, Harry, et al., 2020,^52^ USA	Cross-sectional survey	Physicians	– MBI (EE & DP) – EHR usability (SUS) and Provider task load (PTL)^a^	– EHR-related burnout – Demographic (gender)
19	Melnick, West, et al., 2021,^53^ USA	Cross-sectional survey	Nurses	– MBI (EE & DP) – EHR usability (SUS)^a^	– EHR-related burnout – Demographic (gender) – Professional (years of experience)
20	Olson et al., 2018,^54^ USA	Cross-sectional survey	Physicians	– MBI (22-item) – Mini-Z (10-item)^a^	– EHR-related burnout – Organisational (time, work environment) – Technological (EHR proficiency)
21	Peccoralo et al., 2021,^61^ USA	Cross-sectional survey	Physicians	– MBI (2 items EE, DP) and Mayo Wellbeing Index assessed burnout – EHR frustration & workload (time spent)^a^	– EHR-related burnout – Demographic (female) – Professional (speciality) – Organisational (time after hours) – Technological (vendor)
22	Shanafelt et al., 2016,^16^ USA	Cross-sectional survey	Physicians	– MBI (22-item) – Use of EHR, CPOE & patient portal, satisfaction with EHR and CPOE, perceptions of EHR and patient portals^a^	– EHR-related burnout – Demographic (age, gender) – Professional (speciality) – Organisational (time)
23	Tajirian et al., 2020,^55^ Canada	Cross-sectional survey	Physicians (including fellows and residents)	– Mini-Z (single-item) – Contribution of EHRs toward burnout (single question)^a^	– EHR-related burnout – Organisational (time) – Technological (usability, proficiency, training)
24	Tawfik et al., 2017,^56^ USA	Cross-sectional survey	Physicians & fellows, nurses, and respiratory therapists	– MBI (EE 4-items) – EHR use^a^	– EHR-related burnout – Professional (speciality, years of experience) – Organisational (daily admission, patient acuity)
25	Vehko et al., 2019,^44^ Finland	Cross-sectional survey	Nurses	– Time pressure and psychological distress, – EHR usability factors, – Nurses’ informatics competence^a^	– EHR-related stress – Demographic (age) – Organisational (time pressure) – Technological (usability)
26	Skeff et al., 2022,^62^ USA	Qualitative study	Physicians and graduate medical trainees	– EHR distressing events^a^	– EHR-related stress – Organisational (documentation, billing) – Technological (usability)
27	Callif 2015,^14^ USA	Mixed-methods	Nurses	– Technostress (stress induced by HIT) – Five techno-stressors: techno-overload, techno-uncertainty, techno-complexity, techno-invasion, and techno-insecurity^a^	– EHR-related stress – Organisational (time, complex environment) – Technological (ever-changing, complexity, hardware, software, and networks issues)
28	Hennington 2008,^15^ USA	Mixed-methods	Nurses	– MBI (22-item) – Unified Theory of Acceptance and Use of Technology (UTAUT)^a^	– EHR-related burnout – Organisational (time, caseload) – Technological (IT issues, training)
29	Mazur et al., 2023,^38^ USA	Mixed method survey	Physicians	Quant: – MBI (22-item) – EHR work processes, usability, and workload^a^ – Qual: – EHR-related breakdowns and frustration^a^	– EHR-related burnout – Organisational (documentation, billing) – Technological (usability, Epic)

HIT: health information technology, CPOE: computerised physician order entry, this is part of an EHR

^a^EHR specific measures.

## Results

### Study characteristics

Appendix 6 provides a summary table of study characteristics. A total of 29 studies were included in the systematic review, 26 journal articles,^16,17,38,40–42,44–63^ and three doctoral dissertations.^14,15,43^ Twenty-five studies were quantitative that employed a survey,^16,17,40–61,63^ one was a qualitative study,^62^ and three were mixed-methods studies,^14,15,38^ in which both qualitative and quantitative approaches were used. The majority of included studies were conducted in Northern America (n = 23). Of those, 21 were in the United States including one dissertation conducted in the United States and compared the U.S. findings to India and Germany,^14^ one in Canada,^55^ and one in both the United States and Canada.^17^ Three included studies were conducted in Finland,^44,49,60^ two were conducted in Saudi Arabia,^40,63^ and one in China.^46^ The majority of the study populations were physicians and nurses, in which 18 studies focused on physicians, ten on nurses and three involved both physicians and nurses with other clinicians^42,56,63^ (see Table 2). To clarify, fellows, residents, and house staff are physicians, but these terminologies are often used in the United States depending on their medical degree levels.^66^

### Stress and burnout assessment

Ten out of the 29 studies directly investigated stress related to EHRs, each employing a unique method of assessment. Of those ten studies, one investigated stress related to EHR use using mixed methods,^14^ another study used qualitative method to explore EHR-related distress,^62^ and the other eight were quantitative cross-sectional studies^40,42–44,46,49,57,60^ using a variety of EHR measures. The various EHR measures associated with stress and burnout used in these studies are detailed in Table 2.

Regarding burnout, all 19 studies that measured occupational burnout adopted a quantitative approach. The most common scales used for measuring burnout were the Maslach Burnout Inventory (MBI) (n = 12) and the Mini-Z (n = 7), with one study used both scales.^54^ As both the MBI and the Mini-Z scales measure overall burnout, researchers used EHR metrics alongside these burnout scales to assess the correlation between burnout and specific EHR measures. The MBI is a 22-item scale that involves three domains: (1) emotional exhaustion (EE), (2) depersonalisation (DP), and (3) personal accomplishment (PA). Researchers who used the MBI considered people with high scores on EE or DP as having at least one symptom of burnout. For the 10-item Mini-Z scale, three items were related to EHR use: (1) sufficient time for EHR documentation, (2) the amount of time spent on EHR at home, and (3) proficiency with EHR use. Burnout assessment in the Mini-Z relies on a single-item 5-point scale (using their own definition of burnout). This measure was then correlated with other EHR-related metrics, as depicted in Table 2.

### EHR as a contributor to clinician stress and burnout

All of the reviewed studies indicated that EHR use contributed to clinicians’ stress and subsequent burnout. 19 studies found significant associations between reported burnout rate and EHR-related measures (summarised in Table 2) among physicians,^16,17,38,41,45,47,51,52,54,55,58,61^ nurses,^15,48,50,53,59^ and mixed clinicians; physicians, nurses, respiratory therapists, pharmacists, and others.^56,63^ High levels of stress related to EHR use was reported among physicians,^41,46,47,55,57,62^ among nurses,^40,43,44,48,60^ and similar with both physicians and nurses.^42,63^ In qualitative interviews, physicians,^38,62^ and nurses^14,15^ expressed their stress and frustrations with EHR use. Furthermore, stress related to information systems in a longitudinal study among Finnish physicians during a nine-year follow-up period, showed an increased stress related to information systems trend during the study period demonstrating that the stress level was getting worse over time.^49^

**Figure 2.**
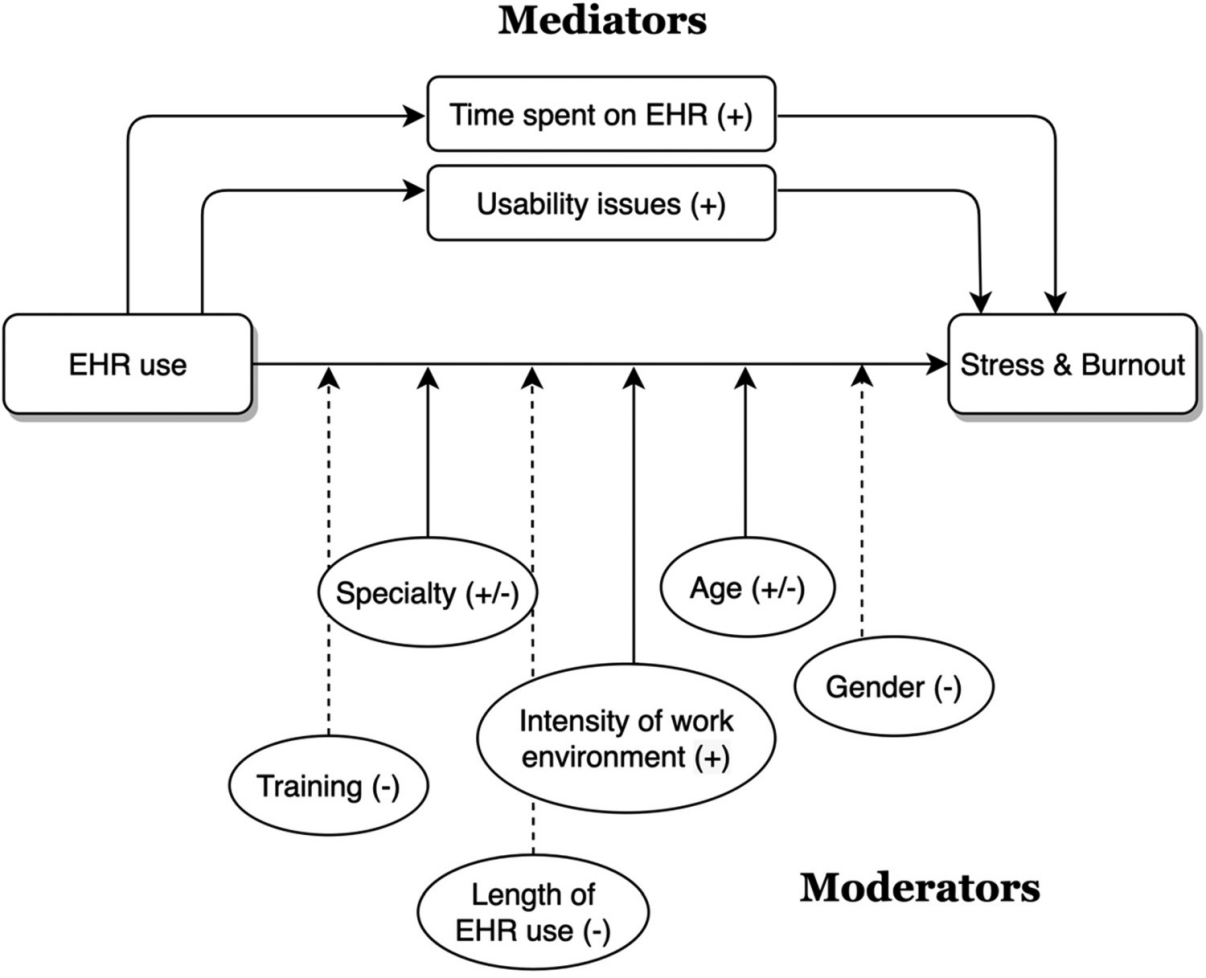
The review findings. The mediator variable explains the reason for a relationship between two independent and dependent variables, EHR use and stress and burnout, respectively, while the moderator variable affects the strength of the relationship between the two variables (independent and dependent). (+) consistent findings (all studies favour a particular conclusion). (−/+) mixed findings (some studies favour a particular conclusion). (−) inconsistent findings (no relationship apparent or no conclusion can be made) . ➜ The arrows show the direction and strength of the relationship.

We developed a model (Figure 2) to summarise the findings of the systematic review. The model provides an explicit answer to the systematic review's two objectives. First, based on a consensus from the studies reviewed, the association between EHR use and clinicians’ stress and burnout was found to be positive (shown in a continuous one-direction arrow). Second, the review identified several contributing factors that mediated or moderated the impact of EHR use on clinicians’ stress and burnout, which will be detailed in the next section.

### Contributing factors to stress and burnout related to EHR use

The contributing factors to clinicians’ stress and burnout related to EHR use are illustrated in Figure 2 as mediators and moderators. Overall, EHR-related stress and burnout were mediated/caused by usability issues and the amount of time spent on the EHR. The factors deemed as moderators, such as age, specialty, and intensity of the working environment, were found to influence the strength of the relationship between EHR use and stress and burnout among clinicians. The strength of these factors was estimated based on the consistency of findings across the reviewed studies (explained under Figure 2). All contributing factors to EHR-related stress and burnout were grouped into four themes: 1) organisational factors, 2) technological factors, 3) demographic factors, and 4) professional factors.

#### Organisational factors: amount of time spent on EHRs, and intensity of the working environment

Amount of time spent on EHRs was the most frequently identified factor in 18 studies contributing to clinician stress and burnout. 13 survey studies found that perceived higher time spent on the EHR was associated with higher levels of stress and burnout among clinicians.^16,17,38,41,45,47,48,54,55,58,59,61,63^ Similarly, based on interviews with physicians^62^ and nurses,^14,15^ perceived time spent on the EHR was a key factor contributing to their stress. Here, physicians and nurses expressed their frustrations with EHR documentation requirements and processes that were perceived to drive them away from spending time with their patients. Particularly frustrating to physicians were the insurance and billing regulations that increased their time spent on EHR.^38,41,62^ In addition, perceived insufficient time for documentation in the EHR,^47,48,54^ and spending extensive time on EHR at home or after hours^47,48,58,61^ were associated with higher rates of burnout. What is more, two studies found that time pressure related to EHR use was associated with high stress related to information systems among physicians,^49^ and psychological distress among nurses.^44^ However, we also found evidence that perceived time spent on data entry may differ from actual time spent on data entry. One study compared self-reported time spent on EHR with the actual time spent, gathered by back-end usage logs, and found an overestimation of the time spent on EHR with a 14-min difference during working hours, and a 5.6-h difference after working hours.^55^ Only one study in Saudi Arabia demonstrated that 83% of clinicians did not have access to the EHR from home,^63^ thus time spent on EHR outside work was insignificant.

Five studies indicated that the intensity of the working environment in inpatient settings and high workloads influenced stress and burnout related to EHR use.^14,15,43,56,63^ The high workload was reported in three studies as a factor causing EHR overload contributing to stress and burnout,^15,43,56,63^ explaining that the higher patient volume and patient acuity, the more EHR workload, driving clinicians to spend more time on EHR after working hours to complete required documentation.^15^ One study found that clinicians who provided tertiary-level of care, and those who cared for patients suspected of having COVID-19 were shown to be at a higher risk of EHR-related burnout.^63^ Nurses reported that issues with EHR use within a busy and complex environment were one of the situations that contributed to their stress.^14^ Two studies identified that work environment was one of the factors contributing to physicians’ burnout;^41,54^ however, the association with EHR use was not measured.

#### Technological factors: EHR usability and training

 EHR usability was a recurring factor reported in 19 studies as contributing to stress and burnout among clinicians. Five studies examined the association between EHR usability and burnout among physicians,^51,52^ and nurses,^50,53,59^ and the findings showed that suboptimal EHR usability scores were strongly associated with higher odds of burnout. In four studies that reported physicians’ burnout related to EHR use, Epic EHR was the most utilised system.^38,47,58,61^ However, only one study measured the association between EHR vendor types and burnout and found no association.^47^ The most frequently cited factors that were associated with clinicians’ stress and burnout related to EHR usability were: poor functionality,^15,38,42,44,49,60,62^ low reliability,^38,44,57,62^ design issues,^15,38,42,55,57,62^ lack of user-friendliness,^45,55^ inflexible order schemes (CPOE),^42^ confusing terminologies,^42^ difficulties with finding^55^ or retrieving information,^40,55^ difficulty editing after the data entry,^40^ network issues,^14^ frequent software updates,^14,43,49,57,60^ lack of adaptability of interface design,^14^ inadequate IT support,^57^ and complexity of the system.^14,43,49^ However, just one study in Saudi Arabia with a low risk of bias showed that despite the reported EHR-related stress and burnout, clinicians’ overall satisfaction with EHR use was relatively high, at 62% on average, with only 34.1% agreeing that EHR use added frustration to their day.^63^ A different study in China showed that physicians’ HIT-related stress was significantly reduced when they used advanced features of EHR as opposed to the basic one.^46^

EHR training was evaluated in six studies;^14,15,40,42,55,58^ however, its effect on EHR-related stress and burnout was uncertain because of contradictory results. Five studies highlighted the benefits of EHR training, but it did not necessarily reduce the level of stress and burnout related to EHR use.^14,15,40,42,55^ For instance, two studies found no significant impact of EHR training on the reduction of stress and frustration among physicians,^42^ and nurses.^14^ It was only associated positively with system familiarity and user satisfaction.^42^ Also, training was perceived as a facilitator, adequately preparing participants for the EHR use.^15,40,55^ On the contrary, one study found that physicians who agreed that their organisation had done a great job with EHR implementation, training, and support were significantly more likely to report lower levels of burnout than those who disagreed.^58^

#### Demographic factors: age and gender

Age was investigated in five studies as a factor that may influence stress and burnout related to EHR use;^16,42,44,48,49^ however, the findings were inconclusive. Three studies found that younger clinicians were more generally satisfied with the EHR system,^16^ tended to be more familiar with it and were frequent users who expressed less stress and frustration when using the EHR than older clinicians.^42,44^ Vehco et al.^44^ found that the increased age of nurses was associated with high levels of psychological distress related to EHR use. In contrast, two studies found no association between age and stress related to the information systems,^49^ and between age and burnout.^48^

Gender was examined in nine studies,^16,17,45,47,51–53,61,63^ but its influence on stress and burnout related to EHR use was uncertain due to inconsistent findings. Four studies found that female clinicians reported higher odds of burnout related to EHR use than males.^16,47,61,63^ Another study showed that male nurses had a higher rate of burnout associated with EHR usability than females.^53^ In contrast, two studies did not find a statistically significant association between gender and burnout related to EHR use.^51,52^ Marckini et al.^17^ and Anderson, Bilal^45^ investigated burnout related to EHR use, and results showed an association between female gender and burnout; however, the role of EHR use in contributing to stress and burnout was not clear.

#### Professional factors: speciality and EHR use experience

Nine studies found that professional speciality influenced the outcomes of stress and burnout associated with the EHR use;^16,42,47,48,51,56,58,61,63^ however, the findings were mixed. Four studies showed that the risk of burnout related to EHR use among physicians varied by medical speciality.^16,51,58,61^ For instance, emergency medicine and anaesthesiology were found to have a higher risk of burnout among physicians than other specialities (e.g., radiology and surgery subspecialities),^16,51^ whereas in the other studies, anaesthesiology was among the specialities with the lowest levels of burnout.^58,61^ Three further studies indicated that burnout was associated with EHR use and EHR-related stress among physicians and nurses.^47,48,63^ However, one study found that physicians were found to have a significantly increased risk of EHR-related burnout in a single study compared to nurses and other healthcare professionals.^63^ Registered nurses tended to have more positive attitudes and perceptions toward EHR use than physicians.^48,63^ Similarly, Ghahramani et al.^42^ found that nurses had higher job satisfaction scores and tended to have more positive perceptions of the CPOE use than physicians. On contrary, Tawfik et al.^56^ found no association between reported burnout related to EHR use in nursing staff.

The length of experience with EHR use might help with system familiarity and reduce EHR-related stressors; however, the evidence found in three studies^40,56,60^ was contradictory and thus inconsistent. Tawfik et al.^56^ found that the prevalence of burnout in Neonatal Intensive Care Units among clinicians with the most longstanding EHR use experience (more than two years) was higher than that among clinicians in units that did not use EHRs. In contrast, AlGahtani et al.^40^ found that EHR-related stress increased with years of clinical experience, and they believed that the transition from paperwork to EHRs may have contributed to senior nurses’ stress. However, a study by Kaihlanen et al.^60^ showed that there was an equal association of SRIS with stress and/or psychological distress for newly graduated nurses (under two years of work experience) and for more experienced nurses (above 2 years of work experience), who were also likely to be more experienced users of the EHR.

## Discussion

### Summary of the main findings

EHR use was a perceived contributor to clinicians’ stress and burnout in hospitals. Usability issues and time spent on EHR were the most significant predictors, but the intensity of the organisational working environment (high workload, patient volume and patient acuity) influenced high EHR-related workload and thereby also contributed to stress and burnout. Among a range of clinicians involved in the review, physicians and nurses were the most studied groups, and the differences in their specialities and duties moderated the levels of stress and burnout related to EHRs. Training and younger age facilitated EHR use but did not reduce perceived levels of EHR-related stress and burnout.

### Integration of findings within the wider empirical literature

The literature on stress and burnout related to EHR use is largely quantitative. This systematic review revealed significant overlap in concepts and inconsistency in how stress and burnout among clinicians using EHRs were assessed. Burnout was measured according to various symptoms including emotional exhaustion, depersonalisation and personal accomplishment from the MBI scale or a single-item (using participants’ own definition of burnout) from the Mini-Z scale.^67,68^ We observed a lack of clarity of burnout symptoms as they are similar to those of the depression.^69,70^ Yet, studies reported that the single-item burnout measure is more specific and practical to measure burnout in healthcare than the 22-item MBI scale, due to its brevity, validity, and ease of administration.^2,7,71^

Almost all studies showing that increased time spent on EHRs was associated with high levels of perceived stress and burnout among clinicians relied on self-reported data. This finding concurs with the wider literature.^72–74^ Only one of the reviewed studies measured both perceived time and actual time spent on EHR and found an overestimation of self-reported time spent on EHR, with a 14-min difference per patient.^55^ There are studies that actually calculated the time spent on EHR including a systematic review of 28 observational studies^75^ and a longitudinal study analysing logs from 65 providers,^76^ in which both findings showed increased time spent on the EHR compared to prior years. Often people tend to report inaccurate estimates of the time length of task durations,^77–79^ but given the subjective nature of stress and burnout, quantitative measures such as time spent on data entry may not accurately reflect impacts on EHR use. This highlights the importance of qualitative research to study stressors associated with clinicians’ experience and time spent using EHRs.

Poor EHR usability was identified as a key factor influencing stress and burnout. This is largely supported by the literature.^24,80^ This refers to a number of design problems reported in this review such as poor performance, non-intuitive user interface, network issues, and difficulty in finding or entering necessary information. EHR usability issues were also reported as a reason for increased time spent on the EHR or causing delays in care, contributing to clinicians’ stress and burnout. Inefficient or counterintuitive design layouts in EHRs can impede the clinicians’ natural workflow leading them to spend extra time and effort navigating the system, and frequently switch between patient care and EHR-related tasks.^81^ This can result in prolonged work hours and a higher cognitive load, causing increased stress and frustration. Additionally, slow system response times, software glitches, or system crashes can halt work, causing delays in care provision. These interruptions not only break the continuity of work but also increase time pressures and induce stress. Better EHR usability leads to higher EHR adoption rates, fewer medical errors, less clinician burnout, improved costs, and improved patient safety.^82^ One of the issues for EHR usability is interoperability (unified, standard format for sharing data between computer systems), which limits customisation opportunities that meaningfully suit local user needs and organisations’ unique workflow and preferences.^83,84^ Simply “one size fits all” solutions will always be challenging because of the countless differences between health organisations, cultures and systems. Studies that developed or redesigned a specific task in their system to fit local users’ needs have measured the task load and found improved EHR usability and decreased mental workload scores among clinicians.^85,86^ This stresses the importance for all stakeholders including healthcare facilities, vendors and policymakers to carefully consider all usability requirements, guided by patient and clinicians’ requirements and feedback, to inform EHR systems and implementation to ensure EHRs are usable and safe.

EHR systems from various vendors might have an impact on EHR usability, but this review has given little attention to the differences between vendors concerning EHR-related stress and burnout. Only Gardner et al.^18^ measured the association between EHR vendors and burnout and found no association. Still, most of the reviewed studies did not specify which EHR systems were being used by healthcare professional, which might be the result of concerns about endorsing or discouraging the use of EHRs from specific companies or manufacturers. Compared to the literature, two studies reported high frustration levels with EHR usability among physicians in critical care settings, which was attributed to the use of Epic EHR systems.^87,88^ The evidence showed that some of the EHR vendors in the USA were not certified or did not perform rigours testing that met the certification requirements of the Office of the National Coordinator for Health Information Technology (ONC) to put the end-users at the center of the process.^89^ Thus, an increased focus on rigorous methods of testing EHRs with frontline clinicians is needed to identify usability challenges and safety hazards in real-world settings. What is more, it is important to incentivise vendors to improve the usability of their systems. There is also the need to be cognisant of the fact that the majority of studies and evidence pertain to the Global North, potentially limiting the generalisability of the findings to other regions.

The evidence in this review showed that the hospital environment is a predictor for EHR-related stress and burnout among clinicians, in which the intensity of the working environment influenced this relationship. Factors associated with the intensity of the working environment were patients’ acuity, high patient volume, and perceived lack of control over the working environment. This suggests that clinicians working in such busier areas in hospitals are at higher risk of burnout associated with EHR use. There was no systematic review that focused exclusively on the association of stress and burnout associated with EHR use in a specific clinical setting. Generally speaking, however, burnout among clinicians specifically physicians and nurses working in hospitals, especially acute and critical care was reported at high levels influenced by the intensity and complexity of the working environment.^1,7,27–31^ Add to this COVID-19 pandemic, which resulted in millions of deaths worldwide, exacerbated this situation. All acute and critical units in hospitals were overwhelmed with patients, which adversely affected clinicians’ wellbeing,^90^ as highlighted in this review. This finding was broadly supported by other studies in this area linking the COVID-19 pandemic with clinicians’ burnout.^91,92^ Therefore, EHR usability should support the different needs of each clinical working setting to reduce associated burdens.

This review showed that age influenced the relationship between EHR use and clinicians’ stress and burnout, in which young clinicians reported fewer stressors related to EHR use, although there were some mixed findings. Frequency of use and system familiarity with EHRs among young clinicians were associated with user satisfaction, which contributed to low levels of stress and frustration. This result accords with a large study on U.S. physician IT use, which revealed that young healthcare workers (less than 40 years old) were the most frequent users of EHRs and other types of technology which was significantly associated with overall satisfaction with EHR use.^93^ It can be argued that young clinicians are highly technology-savvy,^90^ so they have positive and pragmatic attitudes toward it and, accordingly, lower burnout levels than others. However, mixed results suggest that age is not an important factor influencing EHR stress and burnout the same as system usability and/or organisational factors that have a stronger influence on the EHR stress and burnout outcome. Thus, younger age facilitates EHR use and acceptance, but may not be an important factor in reducing stress and burnout related to EHR use.

Clinicians’ speciality was found to have a moderate influence on the relationship between EHR use and clinicians’ stress and burnout. This review identified that physicians and nurses experience stress and burnout related to EHR use at varying levels, yet generally speaking, nurses tended to have more positive attitudes toward EHR use than physicians. This may stem from doctors and nurses being very different types of EHR users, with varying responsibilities and workloads, thus different views about the EHR system and stressors. This is consistent with the findings of previous studies, including systematic reviews.^42,47,48,94–96^ Nonetheless, nurses and physicians shared the same concerns regarding time spent on EHRs at work and home and felt that EHRs took away from their ability to provide bedside care because the documentation demands increased, thereby contributing to additional stress and burnout. This highlights the importance of involving frontline clinicians across the EHR development processes: design, implementation, customisation, evaluation and legislation. In addition, it is important to consider their needs by role, specialities, and subspecialities to improve EHR usability.

Although training was seen as an important element in increasing healthcare workers’ levels of comfort with EHR use and, thus, reducing stress in previous studies,^97–99^ this review does not completely support this evidence. Our findings indicate that the provision of training and sharing of IT knowledge served as facilitators for EHR use but were not necessarily associated with reductions in stressful feelings toward this technology among clinicians. Furthermore, satisfaction with the system did not necessarily reduce or prevent stress and burnout levels. Often EHR vendors bypass EHR usability issues that are identified as some of the main driver of clinicians’ stress and burnout and suggest that health organisations and end-users need more training and an aptitude to learn.^100,101^ Still, a well-designed EHR system requires training tailored to end-users’ requirements, but training should not be used to compensate for poor usability.

### Strengths and limitations

To our knowledge, this is the first systematic review that summarised the current literature investigating stress and burnout among clinicians related to EHR use in hospital settings. This work has followed the systematic review steps guided by PRISMA, to reduce the potential of bias and for more transparent information provided. The evidence was synthesised from mixed methods studies, which included both quantitative and qualitative methods, and relied on peer-reviewed primary studies. The quality of the studies was assessed by appropriate appraisal tools; CASP and MMAT. The information from the reviewed studies was summarised and organised by themes based on a thematic analysis, adding context to quantitative findings.

There are, however, important limitations which are mainly related to the quality of included primary studies. Most used self-reported survey measures of clinician stress and burnout related to EHR use, which have a response bias, thus the lack of rigorous study designs like clinical trials precluded definitive conclusions. It was thus not possible to pool estimates among quantitative studies due to the heterogeneity in the statistics and the assessment tools used for stress and burnout related to EHR use. Furthermore, determining an exact proportion of participants primarily working in the hospital setting proved challenging because of the inclusion of mixed-method studies and the diverse specialties some studies covered. In addition, most of the studies were conducted in the United States (n = 22), which means that generalisability of the review findings can be limited worldwide, as EHR-related stress and burnout might manifest differently in other regions.

### Future implications

Primary stakeholders including healthcare facilities, developers, vendors and policymakers need to find solutions to improve EHR usability to reduce clinicians’ stress and burnout. EHRs need to offer flexibility in workflow design, data entry, and data presentation, and they can be customised to meet each practice environment and clinicians’ specialities needs. What is more, policymakers should eliminate administrative tasks that are not essential to clinicians’ practice to reduce the documentation burden, thus stress and burnout. Therefore, involving clinicians in EHR development to meet their needs may help enhance EHR usability and consequently improve their wellbeing and quality of care. In addition, policymakers should collaborate with clinicians’ experts to develop effective institutional health IT policies.

Stakeholders should note the current trend to be turning resources and attention to better understand the factors that contribute to burnout while identifying ways in which the wellbeing of clinicians can be enhanced. The rationale provided by these studies is that burnout contributing factors can be mitigated or eliminated while the factors that contribute to clinicians’ wellbeing can be reinforced and built upon which would ultimately benefit the healthcare industry as a whole and all the stakeholders associated with it. It is for these reasons that burnout predictors have been the main subject of numerous systematic studies carried out recently as well as this one.

Future research should integrate objective measures with the existing subjective (self-reported) measures to obtain a comprehensive understanding of the impact of EHR use on clinician stress and burnout. On the individual level, physiological indicators such as heart rate variability can offer direct insights into immediate stress responses linked to EHR tasks.^102^ Continuous monitoring can further detail the specific EHR activities causing stress. On the organisational level, metrics such as staff turnover and absence rates, when correlated with EHR usage patterns (e.g., log-in durations, clicks), can illuminate the broader and long-term impacts of EHR use on clinicians’ well-being and efficacy. Further research should also assess the efficiency of multipronged interventions that address the contributing factors to the issue outlined in this review. There is a need for international contributions to this research topic to determine the worldwide prevalence of EHR-related burnout among clinicians and understand the factors that contribute to the problem in different contexts.

## Conclusion

This systematic review showed that EHR use was a perceived contributor to clinicians’ stress and burnout in hospitals. Poor EHR usability and amount of time spent on the EHR were the most significant predictors that mediated EHR-related stress and burnout. The intensity of the working environment also influenced high EHR-related workload and thereby also contributed to stress and burnout. The differences in specialities and duties moderated the levels of stress and burnout related to EHRs, with physicians being the most studied groups. Training and younger age facilitated EHR use but did not reduce perceived levels of EHR-related stress and burnout. To address these issues, there is a need for qualitative research to study stressors associated with clinicians’ experience and time spent using EHRs, as well as the development of EHR systems that are usable and safe, and tailored to meet local user needs and organisations’ unique workflow and preferences. Additionally, there is a need to incentivise vendors to improve the usability of their systems and increase the focus on rigorous methods of testing EHRs with frontline clinicians to identify usability challenges in real-world settings. Finally, given the majority of studies and evidence on Western countries, further research is required to understand the impact of EHRs on clinicians in Non-Western countries.

## Supplemental Material

sj-docx-1-dhj-10.1177_20552076231220241 - Supplemental material for Electronic Health Record Stress and Burnout Among Clinicians in Hospital Settings: A Systematic Review Click here for additional data file. Supplemental material, sj-docx-1-dhj-10.1177_20552076231220241 for Electronic Health Record Stress and Burnout Among Clinicians in Hospital Settings: A Systematic Review by Fatimah Alobayli, Siobhan O’Connor, Aisha Holloway and Kathrin Cresswell in DIGITAL HEALTH

## Abbreviations

ACHD: adult congenital heart disease
CPOE: computerised physician order entry
DP: depersonalisation
EE: emotional exhaustion
EHR: electronic health record
EMR: electronic medical record
HIT: health information technology
MBI: Maslach Burnout Inventory
Mini-Z: Zero burnout program survey
NICU: neonatal intensive care unit
PA: personal accomplishment
