# Supplementary material for: Electronic Health Record Stress and Burnout Among Clinicians in Hospital Settings: A Systematic Review
Source: Digit Health. 2023 Dec 19;9:20552076231220241. doi: 10.1177/20552076231220241 (PMC10734365; doi:10.1177/20552076231220241)
Supplement: sj-docx-1-dhj-10.1177_20552076231220241 - Supplemental material for Electronic Health Record Stress and Burnout Among Clinicians in Hospital Settings: A Systematic Review [file sj-docx-1-dhj-10.1177_20552076231220241.docx]

**Appendix 1: Ovid Databases (Embase, Psychinfo, Ovid Medline)**

| Ovid Research Databases  Embase, PsycINFO, Ovid MEDLINE | | |
| --- | --- | --- |
| # | Search terms | Results |
| 1 | (Nurs* or physician* or doctor* or clinician* or “health practitioner*” or “clinicians*” or “healthcare provider*” or “health care provider*” or “health personnel*” or “allied health” or pharmacist* or “occupational therapist*” or physiotherapist* or midwi*).mp. | 3,805,649 |
| 2 | (Electronic document* or “electronic nursing record” or “nursing information system” or “electronic medical record” or “EMR” or “electronic health record” or EHR or “health information technology” or “HIT” or “patient care information system” or “PCIS” or “computeri?ed Provider Order Entry” or “CPOE” or “Clinical decision support system” or “CDSS” or “Decision support system” or “Electronic prescribing” or “e-Prescribing” or “ePrescribing” or “eHealth” or “e-Health” or “digital health”).mp. | 223,101 |
| 3 | (Burnout or “burn-out” or “burn* out” or stress* or exhaust* or burden* or frustrat* or distress* or cynic* or depersonali?ation).mp. | 3,719,299 |
| 4 | 1 and 2 and 3 | 4,422 |
| 5 | (Conference or note or letter or review or editorial).pt. | 12,946,760 |
| 6 | 4 not 5 | 2,521 |
| 7 | limit 6 to English language | 2,456 |
| 8 | limit 7 to yr=“2000 -Current” | 2,390 |
| 9 | remove duplicates from 8 | 1,624 |

*(.mp)= Multi-purpose of the keyword search, which includes Title, Original Title, Abstract, and Subject Heading.

*(.pt.) = Publication type.

**Appendix 2: CINAHL Database**

| EBSCOhost Research Databases  Database - CINAHL Plus | | | |
| --- | --- | --- | --- |
| # | Query | Limiters/Expanders | Results |
| S1 | Nurs* or physician* or doctor* or clinician* or “health practitioner*” or “clinicians*” or “healthcare provider*” or “health care provider*” or “health personnel*” or “allied health” or pharmacist* or “occupational therapist*” or physiotherapist* or midwi* | Search modes - Find all my search terms | 1,236,481 |
| S2 | Electronic document* or “electronic nursing record” or “nursing information system” or “electronic medical record” or “EMR” or “electronic health record” or EHR or “health information technology” or “HIT” or “patient care information system” or “PCIS” or “computeri?ed Provider Order Entry” or “CPOE” or “Clinical decision support system” or “CDSS” or “Decision support system” or “Electronic prescribing” or “e-Prescribing” or “ePrescribing” or “eHealth” or “e-Health” or “digital health” | Search modes - Find all my search terms | 33,427 |
| S3 | Burnout or “burn-out” or “burn* out” or stress* or exhaust* or burden* or frustrat* or distress* or cynic* or depersonali?ation | Search modes - Find all my search terms | 329,498 |
| S4 | S1 AND S2 AND S3 | Search modes - Find all my search terms | 679 |
| S5 | S1 AND S2 AND S3 | Limiters - Publication Year: 2000-2023; English Language  Search modes - Find all my search terms | 655 |
|  | –133 Duplicates removed manually and by Endnote when combined with Ovid results | | 522 |

**Appendix 3:** **MMAT for Mixed Methods**

| **Category of study designs** | **Methodological quality criteria Responses** | **Author and Date** | | |
| --- | --- | --- | --- | --- |
|  |  | Califf, 2015 | Hennington, 2008 | Mazur et al., 2023 |
| Screening questions (for all types) | S1. Are there clear research questions? | Yes | Yes | Yes |
|  | S2. Do the collected data allow to address the research questions? | Yes | Yes | Can’t tell |
| Qualitative | 1.1. Is the qualitative approach appropriate to answer the research question? | Yes | Yes | Yes |
|  | 1.2. Are the qualitative data collection methods adequate to address the research question? | Yes | Yes | Yes |
|  | 1.3. Are the findings adequately derived from the data? | Yes | Yes | Yes |
|  | 1.4. Is the interpretation of results sufficiently substantiated by data? | Yes | Yes | Yes |
|  | 1.5. Is there coherence between qualitative data sources, collection, analysis and interpretation | Can’t tell | Yes | Can’t tell |
| Quantitative descriptive | 4.1. Is the sampling strategy relevant to address the research question? | Yes | Yes | Yes |
|  | 4.2. Is the sample representative of the target population? | Yes | No | Yes |
|  | 4.3. Are the measurements appropriate? | Can’t tell | Can’t tell | Yes |
|  | 4.4. Is the risk of nonresponse bias low? | Yes | No | Yes |
|  | 4.5. Is the statistical analysis appropriate to answer the research question? | Yes | Yes | Can’t tell |
| Mixed methods | 1. Is there an adequate rationale for using a mixed methods design to address the research question? | Yes | Yes | Yes |
|  | 2. Are the different components of the study effectively integrated to answer the research question? | Yes | Yes | Yes |
|  | 3. Are the outputs of the integration of qualitative and quantitative components adequately interpreted? | Can’t tell | Can’t tell | Can’t tell |
|  | 4. Are divergences and inconsistencies between quantitative and qualitative results adequately addressed? | Can’t tell | Can’t tell | Can’t tell |
|  | 5. Do the different components of the study adhere to the quality criteria of each tradition of the methods involved? | Yes | Yes | Yes |
| **Total score** | | 13/17 | 12/17 | 12/17 |

**Appendix 4: CASP for Qualitative studies**

| **Author and**  **Date** | Q1 –  Was there a clear statement of the research aim? | Q2 – Is a qualitative methodology appropriate? | Q3 –  Was the research design appropriate to address the aims of the research? | Q4 –  Was the recruitment strategy appropriate to the aims of the research? | Q5 –  Was the data collected in a way that addressed the research issue? | Q6 – Has the relationship between researcher and participants been adequately considered? | Q7 –  Have ethical issues been taken into consideration? | Q8 –  Was the data analysis sufficiently rigorous? | Q9 –  Is there a clear statement of findings? | Q10 –  Will the results help locally? | **Total score** |
| --- | --- | --- | --- | --- | --- | --- | --- | --- | --- | --- | --- |
| Skeff et al, 2022 | Yes | Yes | Yes | Yes | Yes | No | Yes | Yes | Yes | Can’t tell | 8/10 |

**Appendix 5: CASP for Survey Designs**

| **Author and**  **Date** | Q1 –  clearly focused issue | Q2 –  recruitment was acceptable | Q3 –  the exposure was accurately measured | Q4 –  the outcome was accurately measured | Q5 –  confounding factors have been accounted for | Q6 –  follow up of the subjects | Q7 –  the results of the study were appropriate | Q8 –  the results of the study  were precise | Q9 –  Do you believe the results? | Q10 –  Can results be applied to the local population? | Q11 –  the study results fit with other available evidence | Q12 –  significance implications for practice | **Total score** |
| --- | --- | --- | --- | --- | --- | --- | --- | --- | --- | --- | --- | --- | --- |
| AlQahtani et al, 2020 | Yes | UC | No | No | Yes | NA | No | UC | No | Yes | No | Yes | 4/12 |
| Almulhem et al., 2021 | Yes | Yes | Yes | Yes | Yes | NA | UC | Yes | Yes | Yes | Yes | Yes | 10/12 |
| Anderson et al., 2022 | Yes | Yes | Yes | Yes | UC | NA | Yes | UC | Yes | Yes | Yes | Yes | 9/12 |
| Chen et al.,  2021 | Yes | Yes | Yes | Yes | No | NA | UC | UC | UC | Yes | Yes | Yes | 7/12 |
| Elliot et al,  2022 | Yes | Yes | Yes | Yes | No | NA | Yes | Yes | Yes | Yes | Yes | Yes | 10/12 |
| Eschenroeder et al., 2021 | Yes | Yes | Yes | Yes | Yes | NA | Yes | Yes | Yes | Yes | Yes | Yes | 11/12 |
| Gardner et al, 2019 | Yes | No | UC | Yes | Yes | NA | Yes | Yes | Yes | Yes | Yes | Yes | 9/12 |
| Gesner et al., 2022 | Yes | Yes | Yes | Yes | UC | NA | Yes | Yes | Yes | Yes | Yes | Yes | 10/12 |
| Ghahramani et al, 2009 | Yes | Yes | UC | Yes | Yes | NA | No | UC | UC | Yes | Yes | Yes | 7/12 |
| Harris et al,  2018 | Yes | No | UC | Yes | Yes | NA | Yes | Yes | Yes | Yes | Yes | Yes | 9/12 |
| Hauer et al,  2018 | Yes | UC | UC | UC | Yes | NA | UC | UC | UC | Yes | Yes | Yes | 5/12 |
| Heponiemi et al, 2017 | Yes | Yes | Yes | UC | Yes | Yes | Yes | UC | Yes | Yes | Yes | Yes | 10/12 |
| Jackson,  2020 | Yes | No | UC | UC | Yes | NA | Yes | UC | Yes | Yes | Yes | Yes | 7/12 |
| Kaihlanen et al., 2021 | Yes | Yes | Yes | Yes | Yes | NA | Yes | Yes | Yes | Yes | UC | Yes | 10/12 |
| Kutney-Lee et al, 2021 | Yes | Yes | Yes | Yes | Yes | NA | Yes | UC | Yes | Yes | Yes | Yes | 10/12 |
| Marckini et al, 2019 | Yes | Yes | UC | UC | Yes | NA | UC | UC | UC | No | Yes | Yes | 5/12 |
| Melnick, Dyrbye et al, 2020 | Yes | Yes | Yes | Yes | Yes | Yes | Yes | UC | Yes | Yes | Yes | Yes | 11/12 |
| Melnick, Harry, et al, 2020 | Yes | Yes | Yes | Yes | Yes | NA | Yes | UC | Yes | Yes | Yes | Yes | 10/12 |
| Melnick, West, et al, 2021 | Yes | Yes | Yes | Yes | Yes | Yes | Yes | UC | Yes | Yes | Yes | Yes | 11/12 |
| Olson et al,  2018 | Yes | Yes | UC | Yes | Yes | NA | Yes | Yes | Yes | Yes | Yes | Yes | 10/12 |
| Peccoralo et al., 2021 | Yes | Yes | Yes | Yes | Yes | NA | Yes | Yes | Yes | Yes | UC | Yes | 10/12 |
| Shanafelt et al, 2016 | Yes | Yes | Yes | Yes | Yes | NA | Yes | Yes | Yes | Yes | Yes | Yes | 11/12 |
| Tajirian et al, 2020 | Yes | Yes | Yes | Yes | Yes | NA | Yes | Yes | Yes | Yes | Yes | Yes | 11/12 |
| Tawfik et al, 2017 | Yes | Yes | Yes | Yes | Yes | NA | Yes | Yes | Yes | Yes | Yes | Yes | 11/12 |
| Vehko et al, 2019 | Yes | Yes | UC | Yes | Yes | NA | UC | UC | Yes | Yes | Yes | Yes | 8/12 |

*UC: unclear, *NA: not applicable.

**Appendix 6: Study characteristics**

| **No** | **Author, Year, Country** | **Research Aims, Theory, Setting & Quality** | **Methods** | **Population** | **Practice setting** | **Limitation** | **Findings** |
| --- | --- | --- | --- | --- | --- | --- | --- |
| 1 | AlQahtani et al, 2020, Saudi Arabia  STRESS STUDY – | Aims: Perceived prevalence of EHR-related stress among nurses and evaluated the determinants of this stress; Setting: Eye Specialist Hospital in Saudi Arabia; Theory: none reported; Quality: Weak. | Ethics: ethical approval granted; Design: cross-sectional survey; Data collection: validated questionnaire from Kroth, Morioka-Douglas [88]; Analysis: regression analysis: Kruskal–Wallis test and Mann–Whitney U test. | Nurses (n=212, response rate 84.8%), mostly females from international countries, who were not trained in the EHR. | Inpatient (recovery room and operation theatre). | The validated instrument the authors used is not the same instrument that has been used by Kroth, Morioka-Douglas [88]. | Half of the nurses working at the hospital perceived stress related to the EHR, with some grading it as severe. Senior nurses and those in the emergency department reported significantly higher EHR related stress. Stress mainly due to incomplete data entry by other colleagues, difficulty searching records, and inability to change data at a later date. |
| 2 | Almulhem et al, 2021, Saudi Arabia  STRESS & BURNOUT STUDY – | Aim: to assess stress and burnout related to the use of EHRs and other HIT tools among HCPs during COVID-19 in Saudi Arabia; Setting: not specified; Theory: none reported; Quality: High | Ethics: ethical approval obtained; Design: cross-sectional survey; Data collection: self-developed survey comprising of 35 items. Mini-Z and HIT-related stress; Analysis: Univariate, bivariate, and multivariate analyses were performed to measure the association between burnout and EHR variables. | 182 participants  Physicians 63%  Nurse 24%  Pharmacist 5%  Others 8% | Governmental and private healthcare organisations:  Primary (19%)  Secondary (25%)  Tertiary (56%) | Relied on only one distribution method, SCFHS’s email database, responses to survey were low, distributing the survey was at the peak of the pandemic in Saudi Arabia with a long data collection period, which may also be related to inadequate responses among HCPs | 50.5% of participants reported a presence of HIT-related stress, and 40.1% reported a presence of burnout. The variables independently associated with burnout were providing tertiary level of care, working with COVID-19 suspected cases, dissatisfaction with EHRs, and agreement with the statement that using EHRs added frustration to the workday. |
| 3 | Anderson et al, 2022, USA  BURNOUT STUDY – | Aim: to examine the prevalence of burnout and identified the contributing factors in gastroenterologists and fellows in training; Setting: not specified; Theory: none reported; Quality: Moderate | Ethics: ethical approval obtained; Design: cross-sectional survey; Data collection: three surveys for three groups of GI physicians; Analysis: Univariable analysis; Kruskal-Wallis tests were used for continuous factors and Pearson χ2 tests were used for categorical variables. | 1,021 participants responded (9.2% response rate) to the first survey, including 756 individuals who completed the MBI survey.  GI physicians and fellows. | GI physicians in rural, urban, suburban settings in the United States. | The first survey was conducted in 2015 before the COVID-19 pandemic. Hence, the findings represent pre-COVID burnout rates, which may underestimate the current prevalence of burnout. | High burnout rate of 49.3%. Factors associated with high burnout were female sex, younger age, shorter duration in practice, considering the EHR non–user-friendly, and increased clinical workload both at work and at home. The level of burnout for fellows was observed to be high (42.7% in survey 2 and 35.3% in survey 3). |
| 4 | Callif et al, 2020, USA  STRESS STUDY – | Aims: test the effects of challenge or hindrance techno-stressors in healthcare; Setting: four hospitals in the U.S.; Theory: Holistic Stress Model; Quality: Moderate. | Ethics: approved by the Research Committee; Design: mixed-methods design; Data collection: interviews with nurses about how they interact with HIT at work, followed by refining the Holistic Stress Model for techno-stressors and testing this new model using online survey data (several existing scales were adapted) from hospital nurses; Analysis: qualitative data analysis, followed by confirmatory factor analysis, common methods bias, and structural equation modelling. | Interviews: 32 practicing nurses and nurse managers from the US.  Surveys: 402 nurses from the US, then 120 nurses from India and 67 nurses from Germany. | Inpatient (acute care). | There was no clear summary of the main findings of the interviews. | The study found a number of factors prevented technostress including the usefulness of HIT, technical support, and managerial involvement. However, several aspects emerged that contributed to technostress such as the unreliability of HIT, its complexity within a busy and complex environment, changes to HIT (hardware, software and networks), which impacted job satisfaction and turnover intention. |
| 5 | Chen et al, 2021, China  STRESS STUDY – | Aim: to extend the existing body of knowledge on HIT by assessing the effects of basic (data-related) and advanced (clinical) HIT features on physician empowerment, stress, and ultimately, job satisfaction; Setting: Chinese hospitals; Theory: none reported; Quality: Moderate | Ethics: ethical approval obtained; Design: cross-sectional survey; Data Collection: questionnaire was administered to measure basic and advanced technologies and physicians’ outcomes; Analysis: used partial least squares a component-based structural equation modelling technique. | 367 physicians completed the survey | Hospitals in China are organised in a 3-tier system (primary, secondary, and tertiary care). | The study did not measure the confounding factors (demographic and organisational) in relation to the study outcomes. | Physicians who used advanced features experienced improvement in all dimensions of physician empowerment and significant reduction in stress. Physicians who used basic technology, however, experienced improvement in fewer dimensions of physician empowerment and no significant change in stress. Except for efficacy, all dimensions of physician empowerment and stress predicted job satisfaction. |
| 6 | Elliot et al, 2022  USA  STRESS STUDY – | Aim: to assess direct and indirect associations between problems with electronic health records (EHRs) and physician distress via problems encountered during the day-to-day practice of medicine and access to social support; Setting: not specified; Theory: none reported; Quality: High | Ethics: ethical approval obtained; Design: cross-sectional survey; Data Collection: 10-item version of the CES-D depression scale, and series of questions about EHR-related problems; Analysis: regression analysis. | 190 physicians (18%) completed the survey. | Physicians across the state of Nevada, who were affiliated with the University of Nevada School of Medicine. | EHR problems were measured as one construct (not segregated). Also, other factors (e.g., demographic or type of work setting) were not measured. | Frequency of EHR problems was positively associated with problems with the day-to-day practice of medicine, and negatively associated with access to social support. Mediation analyses suggest that EHR problems indirectly affect physician distress via problems encountered during the practice of medicine and social support. |
| 7 | Eschenroeder et al, 2021  USA  BURNOUT STUDY – | Aim: to know more about how modifiable dimensions of EHR use relate to burnout and how these associations vary by medical specialty; Setting: not specified; Theory: none reported; Quality: High | Ethics: ethical approval obtained; Design: cross-sectional survey; Data collection: KLAS Arch Collaborative survey to measure the EHR end-user experience, and a single-item from mini-Z for burnout.  Analysis: ordinal logistic regression. | Physicians from all 50 states | Different healthcare organisations. | The response rate for some of the participating organisations is not known, which precludes reporting the overall response rate. Inability to control for sociodemographic variables such as sex, race, and age because they were not included in data collection. | Physicians reporting 5 hours weekly of after-hours charting were twice as likely to report lower burnout scores compared to those charting 6 hours. Physicians who agree that their organisation has done a great job with EHR implementation, training, and support were also twice as likely to report lower scores on the burnout survey question compared to those who disagree |
| 8 | Gardner et al, 2019, USA  BURNOUT STUDY – | Aims: 1) determine the prevalence of burnout symptoms and HIT-related stress and 2) quantify the association of HIT-related stress with burnout among physicians; Setting: general healthcare in Rhode Island; Theory: none reported; Quality: High. | Ethics: deemed exempt by ethics board; Design: cross-sectional; Data collection: online survey in 2017 of all physicians in one U.S. state; Burnout measured using a single item from the Mini-Z a 10-item instrument developed from the Physician Work Life Study; Analysis: Univariable statistics, bivariable chi-square tests, multivariable logistic regression, ordered logit model with sensitivity analysis. | Licensed physicians (n=1792, response rate 42.7%) in Rhode Island. | Mixed settings  Inpatient: 32.3%  Outpatient: 67.6% | Responses were not anonymous.  Also, authors measured the association between EHR vendors and burnout and found no association, but they didn't provide any statistical data related to vendors in the study. | Among those who used the EHR (91%), 70% reported HIT-related stress. The highest prevalence was in primary care specialties. Factors contributing to burnout included: 1) poor/marginal time for documentation, 2) excessive time on EHRs at home, and 3) those who agreed that EHRs add to their daily frustration. |
| 9 | Gesner et al, 2022, USA  BURNOUT STUDY – | Aim: to understand the relationship between documentation burden and clinician burnout syndrome in nurses working in direct patient care; Setting: not specified; Theory: Roy’s adoption model; Quality: High | Ethics: IRB approval obtained  Design: cross sectional survey; Data collection: 22-item MBI, and SUS survey tools; Analysis: A Pearson correlation test to test the association between two variables. | 69 nurses included.  Registered nurses who work in a direct patient care role and document in the EHR with at least1year of experience were included. | Different settings, and the majority was inpatient settings. | Low number of the study participants.  The demographic factors were not correlated with burnout. | Documentation burden has a weak to moderate correlation to clinician burnout syndrome. Furthermore, poor usability of the EHR is also associated with documentation burden and clinician burnout syndrome. |
| 10 | Ghahramani et al, 2009, USA  STRESS STUDY – | Aims: evaluate perception of attending physicians, house staff, nurses, and senior medical students regarding efficiency and ease of CPOE, stress in work place, system  training, and user satisfaction; Setting: Milton S. Hershey Medical Center; Theory: none reported; Quality: Moderate. | Ethics: ethical approval granted; Design: not reported; Data collection: online survey with 110 questions on CPOE use, Perceived Stress Scale [89], job performance and satisfaction including a Generic User Interface Question [90]; Analysis: Total scores, Cronbach’s Alpha, One-Way ANOVA with Tukey’s Studentized test, t-tests, Pearson’s correlation Coefficient performed via SPSS. | 862 regular users of the CPOE system - 209 nurses, 178 attending physicians,  78 fellows, 179 upper-level residents, 83 interns, and 135  medical students. | Hospital/inpatient | The survey was long (110 Qs), which might have affected the quality of the responses. | 413 respondents (47.9 % response rate). Those younger in age were more familiar with the CPOE system, used it more often, and were more satisfied with it. Interns and residents were the most satisfied groups with the CPOE, while attending physicians expressed the least satisfaction. Attending physicians and fellows found the CPOE least user friendly compared with  other groups, and also tended to express more stress and frustration with it. |
| 11 | Harris et al, 2018, USA  BURNOUT STUDY – | Aims: estimate the association between EHR-related stress and burnout among APRNs and describe their perceptions about HIT; Setting: Healthcare in Rhode Island; Theory: none reported; Quality: High. | Ethics: deemed exempt by ethics review board; Design: cross-sectional; Data collection: electronic survey on HIT use, Burnout measured using single Mini-Z item a 10-item instrument developed from the Physician Work Life Study; Analysis: Bivariable chi-square and Fisher's exact tests, logistic regression and multivariable logistic regression via SPSS. | Advanced practice registered nurses (APRNs) in one U.S. state, n=371 (response rate 31%). | Mixed setting  Inpatient: 67.6%  Outpatient: 32.4% | Survey was not anonymous, which might have contributed to underreporting of the prevalence of burnout among respondents. | 73 APRNs reported at least one symptom of burnout. Of these, 34 (46.6%) were Family/Individual APRNs and 16 (21.9%) were Adult/Gerontology APRNs.  Those who use EHRs, 64 (19.3%) reported spending a moderately high to excessive amount of time on their EHR at home, 165 (50.1%) agreed or strongly agreed EHRs add to their daily frustration, and 97 (32.8%) reported insufficient time for documentation. |
| 12 | Hauer et al, 2018, USA  BURNOUT STUDY – | Aims: assess current levels of physician satisfaction and burnout; Setting: multiple medical specialities; Theory: none reported; Quality: Weak. | Ethics: not reported; Design: cross-sectional; Data collection: online survey using the full Mini-Z questionnaire (48 questions), administered to American Medical Association (AMA) members in one U.S. state; Analysis: not reported. | 1,165 physicians across multiple specialities (8.86% response rate). | Mixed settings  Not specified | Methods were not explained in detail. Statistical analysis was not reported.  Ethical approval was not reported.  References provided for the survey links can’t be accessed. | One of the three categories of physician burnout was EHR / documentation time. 65% of clinicians agreed or strongly agreed that using an EHR adds frustration to their day. 42% who spent 0-2 hours working at home on the EHR report being frustrated with it, which is almost half the frustration level reported by physicians who spend >8 hours on the EHR outside of work. |
| 13 | Hennington, 2008, USA  BURNOUT STUDY – | Aims: develop an understanding of nurses lived experiences using an electronic medical record (EMR); Setting: large urban hospital; Theory: Unified Theory of Acceptance and Use of Technology (UTAUT); Quality: Moderate | Ethics: institutional review board approval; Design: mixed methods; Data collection: interviews with nurses (questions based on UTAUT) and direct non-participant observation of nurses using EMRs followed by an electronic survey (burnout measured using Maslach’s Burnout Inventory), followed by a qualitative case study; Analysis: inductive and deductive analysis followed by partial least squares for structural equation modelling. | 23 nurses and 4 nurse managers were interviewed, 65 nurses completed the survey. | Hospital/inpatient | Small sample size in the survey (n-65). No descriptive tables or graphs of the survey. The interpretation of the survey results was not clear. | Some predictors of EMR usage included performance expectancy, effort expectancy, social influences, facilitating conditions and caseloads. Outcome usage included role conflict and role overload. |
| 14 | Heponiemi et al, 2017, Finland  STRESS STUDY – | Aims: examine the 9-year longitudinal development of stress levels related to information systems (SRIS) among Finnish physicians; Setting: not specified; Theory: none reported; Quality: High | Ethics: ethical approval obtained; Design: longitudinal survey in 2006, 2010 and 2015; Data collection: wave 1, 2 and 3 used a mix of web-based and postal questionnaires; Analysis: GLM repeated measures analysis. | 2841 physicians responded in wave 1, 1705 responded in wave 2, 1462 physicians responded in wave 3. | Mixed  Inpatient: 32%  Outpatient: 15%  Private sectors: 9.5%  Others: NR |  | SRIS increased during the study period. The increase was most pronounced in primary care, whereas in hospitals SRIS did not increase between 2010 and 2015. SRIS increased more among those in a leadership position. On-call duties and high time-pressures were associated with higher SRIS levels during all waves. |
| 15 | Jackson, 2020, USA  STRESS STUDY – | Aims: determine the prevalence of technostress in among hospital nurses and examine the relationship between technostress and nurses personality traits; Setting: 14 hospitals under one system in Florida; Theory: Transactional Model of Stress and Coping; Quality: Moderate | Ethics: ethical approval granted; Design: non-experimental; Data collection: a convenience sample of surveys from nursing working with EHR on a daily basis.; Analysis: bivariate and multivariate regression analysis. | Hospital nurses n=157 (2.7% response rate) | Inpatient (acute and critical care units). | Small sample size n=157 of 5,788 from 14 hospitals. | Nurses experience technostress at a very high level within the hospital setting. The highest rate of technostress was within job insecurity 31%, followed by techno-invasion 22.9%, techno-complexity 13.4%, techno-uncertainty 3.2%, and techno-overload 1.9%. |
| 16 | Kaihlanen et al, 2021, Finland  STRESS STUDY – | Aim: to examine whether SRIS and nursing informatics competence are associated with stress and psychological distress in newly graduated nurses (NGNs) and experienced nurses; Setting: not specified; Theory: none reported; Quality: High | Ethics: ethical approval obtained; Design: cross-sectional survey; Data collection: survey tools measuring stress, psychological distress, SRIS, nursing informatics competence; Analysis: multiple linear regression analysis. | NGNs (n = 712) with less than two years of work experience and experienced nurses (n = 1226) with more than two years of work experience | Mixed types of settings inpatients and outpatients. | SRIS was measured with only two items with a Cronbach’s alpha value that was low. | SRIS was associated with stress/psychological distress for both NGNs and experienced nurses. Higher nursing informatics competence was associated with lower stress and psychological distress in NGNs, but not among experienced nurses |
| 17 | Kutney-Lee et al, 2021, USA  BURNOUT STUDY – | Aims: examine the associations between EHR usability and nurse job (burnout, job dissatisfaction, and intention to leave) and surgical patient (inpatient mortality and 30-day readmission) outcomes; Setting: hospitals in 4 US states; Theory: none reported; Quality: High | Ethics: ethical approval granted; Design: retrospective, cross-sectional study; Data collection: secondary data sources collected between 2015 and 2016, including: (1) the American Hospital Association (AHA) Annual Survey of Hospitals, (2) the AHA Healthcare Information Technology (IT) database, (3) patient discharge abstracts obtained from state agencies, and (4) the RN4CAST-US nurse survey; Analysis: logistic regression models | 12,004 RNs and 1,281,848  patients embedded in 343 hospitals across the 4 states. | Inpatient (acute and critical care units). |  | Nurses who worked in hospitals with poorer EHR usability had significantly higher odds of burnout, job dissatisfaction and intention to leave compared with nurses working in hospitals with better usability. Surgical patients treated in hospitals with poorer EHR usability had significantly higher odds of inpatient mortality and 30-day readmission compared with patients in hospitals with better usability. Comprehensive EHR adoption was associated with higher odds of nurse burnout. |
| 18 | Marckini et al, 2019, Canada and United States  BURNOUT STUDY – | Aims: To determine burnout in adult congenital heart disease (ACHD) specialists by assessing stress associated with EHRs; Setting: a variety of practice settings and environments; Theory: none reported; Quality: Weak | Ethics: ethical approval granted; Design: Electronic survey study of ACHD providers; Data collection: Burnout was measured using the Maslach Burnout Inventory (MBI); Analysis: Chi square and Wilcoxon Rank Sum tests | 110 ACHD specialists responded to the survey (28.7% response rate). The majority worked in an academic medical center (n=88, 80.7%). | Mixed  Not specified, but the majority (80.7%) worked in hospital/inpatient. | Llimited responses from ACHD providers in Canada (n = 5). Shortage of data provision and interpretation (no tables too). | 40% (n = 44) ACHD specialists met the criteria for burnout, and they strongly disagreed that a reasonable amount of time is spent on clerical tasks related to direct or indirect patient care. Female physicians were found to have higher incidence of emotional exhaustion than male physicians. There was strong disagreement that EHRs improved efficiency or that the patient portal improved patient care. Physicians >55 years old had a higher perception of personal accomplishment than their younger peers. |
| 19 | Mazur et al, 2023, USA  BURNOUT STUDY – | Aim: to understand the key factors contributing to hospitalists’ burnout and identify key priorities for improving hospitalists’ workplace; Setting: academic medical centre and a community hospital; Theory: socio-technical model; Quality: Moderate | Ethics: ethical approval obtained; Design: mixed-method methodology; Data collection: Quan: 22-item Maslach Burnout Inventory, and ST workplace factors including EHR. Qual: contextual inquiry-data-collection (field observation) and modelling activities, and focus-group led validation and prioritisation of ST factors to be addressed through system-wide improvements; Analysis: t-test (two-tailed) for the quantitative, and an Affinity Model for the qualitative analysis. | 58 hospitalists with a response rate of 68%. | Inpatient hospital setting | Sample selected by the hospitalist leadership. | 76% of hospitalists reported elevated levels on at least one sub-scale of the MBI. During CIs, key breakdowns were reported in relationships, communication, coordination of care, work processes in EHR, and physical space. Using data from CIs, an affinity diagram was developed. |
| 20 | Melnick, Dyrbye et al, 2020, USA  BURNOUT STUDY – | Aims: to describe and benchmark physician-perceived electronic health record (EHR) usability and evaluate the association with professional burnout among physicians; Setting: not specified; Theory: none reported; Quality: High | Ethics: ethical approval granted; Design: secondary analysis; Data collection: cross-sectional survey of US physicians from all specialties from October 2017 to March 2018, Burnout was measured using the Maslach Burnout Inventory; Analysis: Kruskal-Wallis or X^2^ tests, and multivariable analysis using linear and logistic regression. | 870 physicians (69.6%) completed a sub-survey of EHR usability. 74.4% were non-primary care physicians. | Mixed  Not specified, but the majority worked in hospitals/inpatients. |  | 397 of 864 (45.9%) had at least 1 symptom of burnout. Mean SD SUS score was 45.9+/-21.9. A score of 45.9 is in the bottom 9% of scores across previous studies and categorized in the “not acceptable” range or with a grade of F. EHR usability scores were independently associated with the odds of burnout with each 1 point more favorable SUS score associated with a 3% lower odds of burnout. |
| 21 | Melnick, Harry, et al, 2020, USA  BURNOUT STUDY – | Aims: determine the relationship between physician perceived EHR usability and workload by specialty and evaluate for associations with professional burnout; Setting: not specified; Theory: none reported; Quality: High | Ethics: ethical approval granted; Design: cross-sectional survey; Data collection: survey of US physicians from October 2017 to March 2018, Burnout was measured using the Maslach Burnout Inventory; Analysis: Wilcoxon rank sum test or X^2^ test, univariable and multivariable analysis using linear and logistic regression. | 5197 physicians n=848 (67.8%) completed a sub-survey of EHR usability. | Mixed  Not specified, but the majority worked in hospitals/inpatients |  | A strong association was observed between EHR usability and workload among US physicians, with more favorable usability associated with less workload. Both outcomes were associated with the odds of burnout, with task load acting as a mediator between EHR usability and burnout. |
| 22 | Melnick, West, et al, 2021, USA  BURNOUT STUDY – | Aims: to measure nurse-perceived electronic health records (EHR) usability with a standardized metric of technology usability and evaluate its association with professional burnout; Setting: not specified; Theory: none reported; Quality: High | Ethics: ethical approval granted; Design: cross-sectional survey; Data collection: random sample of US nurses was conducted in November 2017, Burnout was measured using the Maslach Burnout Inventory; Analysis: Kruskal-Wallis or X^2^ tests, and multivariable analysis using linear and logistic regression. | 8,638 nurses (9.9%) completed the survey. | Mixed  Inpatient: 78%  Outpatient: 22% |  | 42.0% were determined to be burnt out. Nurses rated the usability of their current EHR in the low marginal range of acceptability using a  standardized metric of technology usability. EHR usability scores were associated with burnout with each 1 point more favorable SUS score and associated with a 2% lower odds of burnout. |
| 23 | Olson et al, 2018, USA  STRESS & BURNOUT STUDY – | Aims: identify remediable stressors associated with burnout and to compare performance of the Mini‐Z's single‐item burnout metric against the 22‐item MBI; Setting: academic medical centre; Theory: none reported; Quality: High | Ethics: exempted from IRB review under federal regulation; Design: cross‐sectional study; Data collection: prevalence of burnout was determined with the MBI and the Mini‐Z survey; Analysis: chi‐square test, one‐way ANOVA, and multivariate logistic regressions. | 557 responded anonymously (44% completion rate). 475 were included; academic faculty (372), hospital employed (52), and private practitioners  (81). | Mixed  Not specified | The majority of the respondents are academic, in which bias affected the results. | Prevalence of burnout via the MBI was 56.6%. Predictors of burnout were poor control over workload, inefficient teamwork, insufficient documentation time, hectic‐chaotic work atmosphere, lack of value‐alignment with leadership, and excessive electronic medical record time at home. Academic faculty experienced more burnout than private practitioners. Odds of burnout associated with stressors were generally concordant via Mini‐Z's burnout metric versus the MBI. |
| 24 | Peccoralo et al, 2021, USA  BURNOUT STUDY – | Aim: To identify specific thresholds of daily electronic health record (EHR) time after work and daily clerical time burden associated with burnout in clinical faculty; Setting: Mount Sinai Health System, a large academic medical  centre with 7 hospital sites in New York City; Theory: none reported; Quality: High | Ethics: ethical approval obtained; Design: cross-sectional survey; Data collection: 2 validated instruments to measure burnout; Well-Being Index (WBI) and the Maslach Burnout Inventory (2 subscales). EHR and clerical work items were modified from the Mini-Z; Analysis: multivariable logistic and linear regression models. | 1781(42.9%) participated in the survey. The study focused on 1346 (75.6%) faculty who spent some time on patient care duties. | Mixed inpatient and outpatient settings. | Three departments  used different EHRs, some of which have integrated  dictation systems, which could have affected their EHR and clerical burden and ultimately impacted their level of burnout. | EHR frustration, spending >90 minutes on EHR-outside the workday by self-report and >1 hour of self-reported clerical work/day were associated with burnout. Reporting that one’s practice unloads clerical burden and higher resilience scores were negatively associated with burnout. |
| 25 | Shanafelt et al, 2016, USA  BURNOUT STUDY – | Aims: evaluate the relationship between the electronic environment, clerical burden, and burnout in US physicians; Setting: physicians across all specialties in the US; Theory: none reported; Quality: High | Ethics: not reported; Design: a cross-sectional; Data collection: Burnout measured using the Maslach Burnout Inventory; Analysis: Kruskal-Wallis or X^2^ tests, and multivariable regression analysis. | Of 6375 responding physicians in active practice, 5389 (84.5%) used EHRs, and 82.5% reported using CPOE. 76.6% were non-primary care physicians. | Mixed  Not specified, but the majority worked in hospitals/inpatients | Gender factor showed significant association to burnout (table 4) but not explained in the paper. | Physicians who used EHRs and CPOE had lower satisfaction with the amount of time spent on clerical tasks and higher rates of burnout. Physicians who used EHRs or CPOE were less likely to be satisfied with the amount of time spent on clerical tasks after adjusting for age, sex, specialty, practice setting, and hours worked per week. Use of CPOE was also associated with a higher risk of burnout after adjusting for these same factors. |
| 26 | Skeff et al, 2022, USA  STRESS STUDY – | Aim: to assess how the EHR induces distress in physicians and its impact on their  professional behaviours; Setting: two healthcare organisations in Northern California; Theory: action research; Quality: High | Ethics: ethical approval obtained; Design: qualitative design; Data collection: semi-structured interviews; Analysis: grounded theory approaches | 50 physicians and graduate medical trainees | Mixed |  | EHR-related distress affecting professional activities. Five main themes emerged from our analysis: system blocks to patient care; poor implementation, design, and functionality of the EHR; billing priorities conflicting with ideal workflow and best-practice care; lack of efficiency; and poor teamwork function |
| 27 | Tajirian et al, 2020, Canada  BURNOUT STUDY – | Aims: (1) identify the extent of burnout and the perceived contribution of the EHR toward it; (2) identify significant contributors of burnout and EHR-related burnout; (3) explore differences between physicians and learners among factors previously identified as contributing to EHR-related burnout; and (4) compare self-reported perceptions on EHR usage metrics using log data; Setting: academic mental health hospital; Theory: none reported; Quality: High | Ethics: ethical approval obtained; Design: cross-sectional survey design; Data collection: electronic survey, Burnout measured using Mini-Z survey, contribution of EHRs toward burnout was measured by a single question on a 4-point scale, and open-ended survey responses about the experience with the EHR; Analysis: Chi-square and Fisher exact tests. | 176 physicians and 32 learners (fellows and residents) responded.  The response rates were 43.2% for physicians (full-time: 75% and part-time: 10%) and 47.7% for  learners (fellows: 86% and residents: 40%). | Mixed settings  Not specified |  | A total of 25.6% (45/176) of practicing physicians and 19% (6/32) of learners reported having one or more symptoms of burnout, and 74.5% (155/208) of all respondents who reported burnout symptoms identified the EHR as a contributor. Lower satisfaction and higher frustration with the EHRs were significantly associated with perceptions of EHR contributing toward burnout. Physicians’ and learners’ experiences with the EHR, gathered through open-ended survey responses, identified challenges around the intuitiveness and usability of the technology as well as workflow issues. Metrics gathered from back-end usage logs demonstrated a 13.6-min overestimation in time spent on EHRs per patient and a 5.63-hour overestimation of after-hours EHR time, when compared with self-reported survey data. |
| 28 | Tawfik et al, 2017, USA  BURNOUT STUDY – | Aims: test the relation between provider burnout prevalence and organisational factors; Setting: 41 NICUs in California; Theory: none reported; Quality: High | Ethics: ethical approval obtained; Design: cross-sectional survey; Data collection: Burnout measured using a 4-item questionnaire based on the Maslach Burnout Inventory; Analysis: 2-tailed t-test, pearson’s correlation coefficients, univariable regressions, sensitivity analysis. | 1934 respondents (70% response rate), physicians, nurse practitioners, registered nurses, and respiratory therapists. Most respondents were nurses (75%), and 84.8% were females. | Inpatients (NICUs) |  | Overall burnout prevalence was 26.7% ± 9.8%. Burnout was most prevalent in NICUs with high patient volume and electronic health records. Nursing burnout was more sensitive to organizational differences than physician. |
| 29 | Vehko et al, 2019, Finland  STRESS STUDY – | Aims: explores the associations of EHR usability factors and nurses’ informatics competence factors with self-reported time pressure and psychological distress among registered nurses; Setting: not specified; Theory: none reported; Quality: Moderate | Ethics: ethical approval obtained; Design: nationwide survey conducted in 2017; Data collection: stress measured via the Harris stress index and psychological distress via four items from the General Health Questionnaire (GHQ) and a four-factor model of EHR-related usability factors; Analysis: Analyses of covariance (ANCOVA) were conducted in three steps. | 3607 nurses responded (5% men) — a response rate of 12% | Mixed  Not specified, but half (54%) of the RNs worked in hospitals/inpatients | Result section is brief, not described in detail. | Unreliability and poor user-friendliness of EHRs seem to be prominent sources of time pressure and psychological distress among registered nurses. |
